# Gut Microbiota and Adipose Tissue Microenvironment Interactions in Obesity

**DOI:** 10.3390/metabo13070821

**Published:** 2023-07-05

**Authors:** Congcong Wang, Zihan Yi, Ye Jiao, Zhong Shen, Fei Yang, Shankuan Zhu

**Affiliations:** 1 Chronic Disease Research Institute, The Children’s Hospital, and National Clinical Research Center for Child Health, School of Public Health, School of Medicine, Zhejiang University, Hangzhou 310058, China; ccwang@zju.edu.cn (C.W.); 3180102744@zju.edu.cn (Z.Y.); 3190100551@zju.edu.cn (Y.J.); 2 Department of Nutrition and Food Hygiene, School of Public Health, School of Medicine, Zhejiang University, Hangzhou 310058, China; 3 Department of Colorectal Surgery, Affiliated Hangzhou Dermatology Hospital, Zhejiang University School of Medicine, Hangzhou 310009, China; legandsky@gmail.com

**Keywords:** gut microbiota, metabolites, adipose tissue microenvironment, obesity

## Abstract

Obesity is an increasingly serious global health problem. Some studies have revealed that the gut microbiota and its metabolites make important contributions to the onset of obesity. The gut microbiota is a dynamic ecosystem composed of diverse microbial communities with key regulatory functions in host metabolism and energy balance. Disruption of the gut microbiota can result in obesity, a chronic metabolic condition characterized by the excessive accumulation of adipose tissue. Host tissues (e.g., adipose, intestinal epithelial, and muscle tissues) can modulate the gut microbiota via microenvironmental interactions that involve hormone and cytokine secretion, changes in nutrient availability, and modifications of the gut environment. The interactions between host tissues and the gut microbiota are complex and bidirectional, with important effects on host health and obesity. This review provides a comprehensive summary of gut microbiota changes associated with obesity, the functional roles of gut microbiota-derived metabolites, and the importance of the complex interactions between the gut microbiota and target tissues in the pathogenesis of obesity. It places particular emphasis on the roles of adipose tissue microenvironment interactions in the onset of obesity.

## 1. Introduction

Obesity, a widespread public health problem, has serious negative effects on quality of life and well-being worldwide. According to the World Health Organization, obesity rates have doubled since 1975, such that it currently affects more than 650 million adults globally [1]. This alarming trend is attributed to numerous factors, including changes in dietary habits and physical activity levels, as well as sedentary lifestyles; thus, it constitutes a primary public health concern in many countries.

The gut microbiota, a large community containing trillions of bacteria, viruses, and other microorganisms, plays a key role in the progression of obesity [2]. There is substantial evidence regarding its essential roles in energy regulation, whereby it facilitates nutrient uptake, metabolism, and storage [3]. Moreover, inherent dysbiosis within the gut microbiota has been implicated in the pathogenesis of obesity and associated metabolic morbidities [4].

In recent years, extensive research (e.g., large-scale population studies, animal models, and cellular cultures) has improved the overall understanding of the role of the gut microbiota in obesity. A pivotal study, published in 2006, demonstrated divergent gut microbiota patterns between morbidly overweight individuals and their lean counterparts, providing initial support to the notion that the gut microbiota is involved in the onset of obesity [3]. A subsequent study, published in 2013, revealed that the transfer of gut microbiota from overweight mice to lean cagemates resulted in weight gain; this finding highlighted the critical regulatory role of the gut microbiota in energy homeostasis [5]. A study published in 2014 showed that the consumption of specific probiotics could improve weight loss and enhance metabolic function among obese individuals, emphasizing the potential for gut microbiota modulation to be used in clinical treatment [6]. Since then, there have been efforts to identify the roles of gut microbiota-derived metabolites, including bile acids, short-chain fatty acids (SCFAs), and amino acids, in the development of obesity. A study published in 2016 demonstrated that the levels of specific gut microbiota-derived metabolites were increased in individuals with obesity and metabolic-related diseases, thereby strengthening the link between the gut microbiota and obesity [7]. These findings imply that the gut microbiota and its metabolites are key factors with critical effects on obesity pathogenesis.

This review provides a comprehensive synopsis of the dynamic changes and functional properties of the gut microbiota and their metabolites during the onset and progression of obesity; it also explores underlying mechanisms that govern the interactions between the microbiota and target tissues in obesity, with particular emphasis on the influential role of the microbiota in establishing the adipose tissue microenvironment. The main objective of this review is to provide a detailed framework for the current understanding of the importance of the gut microbiota and its metabolites in the context of obesity, thereby offering a valuable reference for future research endeavors focused on microbiota-related human obesity.

## 2. The Association between the Gut Microbiota and Obesity

There is considerable evidence of a strong association between obesity and the gut microbiota; significant changes in gut microbiota composition have been observed in obese individuals. Table 1 provides an overview of the common obesity-related changes in microbiota [8,9,10]. Several animal and human studies have demonstrated microbial changes in obesity condition. For example, Turnbaugh et al. [2] compared obese and lean individuals in mice and human volunteers and observed a reduction in Bacteroidetes and a proportional increase in Firmicutes in the obese group. They suggested that the obese microbiome has an increased capacity to harvest energy from the diet and that this trait was transmissible, indicating that the gut microbiota is an additional contributing factor to the pathophysiology of obesity. Similarly, Geurts et al. [11] found a significant higher abundance of Firmicutes, Proteobacteria, and Fibrobacteres phyla in db/db mice compared to lean mice. Furthermore, Gophna et al. [12] showed that the presence of a bacterial genus named Oscillospira, which belongs to Clostridial cluster IV, is reduced in obesity as well. They inferred that Oscillospira species were butyrate producers, and at least some of them have the ability to utilize glucuronate to help explain the observation that the presence of this genus is reduced. Treatments for obesity also lead to changes in the gut microbiota. In a dietary intervention trial for obese individuals, Remely et al. [13] observed that Lactobacillus, Clostridium cluster IV, Faecalibacterium prausnitzii, and Archaea increased during intervention, while Clostridium cluster XIVa showed a decreased abundance, indicating a marked change of gut bacterial composition through dietary intervention. Furet et al. [14] profiled gut microbita from fecal samples in control subjects and obese individuals before and after Roux-en-Y gastric bypass (RYGB) surgery, one of the most efficient procedures for the treatment of morbid obesity. They found the presence of Bacteroides/Prevotella group was lower in obese subjects, which was negatively correlated with corpulence.

Changes in gut microbiota composition and functionality have been implicated in the onset and progression of obesity via modulation of energy metabolism, insulin sensitivity, and inflammatory signaling pathways [18]. Nevertheless, further research is needed to gain comprehensive insights into the changes in the gut microbiota and microbiota-associated metabolites in the context of obesity, with the goal of resolving the complex interactions between the gut microbiota and obesity.

## 3. Gut Microbiota-Derived Metabolites Associated with Obesity

The gut microbiota has a central role in the biosynthesis of various metabolites, including bile acids, amino acids, SCFAs, and neurotransmitters. These metabolites have extensive effects on energy metabolism, insulin sensitivity, and the onset of obesity and related metabolic disorders [19]. There remains a considerable lack of understanding regarding the gut microbiota’s effects on host metabolism and body weight, but the past decade has yielded important mechanistic insights into these interactions [20]. Recent research has revealed both direct and indirect effects of the gut microbiota on calorie absorption and host metabolic pathways through the production or modification of microbial or host-derived compounds [21]. This review provides a comprehensive overview of the main evidence concerning the microbiota-derived metabolites involved in obesity, as shown in Table 2.

### 3.1. Bile Acids

Bile acids, a class of steroid compounds, are synthesized in the liver and stored in the gallbladder. Upon food intake, these molecules are released into the small intestine where they play important roles in the digestion and absorption of dietary fat, as well as fat-soluble vitamins. Bile acids also function as signaling molecules, with key contributions to systemic metabolic maintenance. These contributions are facilitated by their interactions with various nuclear receptors, including (but not limited to) the farnesoid X receptor [35], vitamin D receptor [36], pregnane X receptor [37], and androstane receptor [38], as well as membrane receptors (e.g., Takeda G protein-coupled receptor 5 [39]). These interactions regulate the secretion of key factors such as peptide YY (PYY), glucagon-like peptide-1 (GLP-1), and fibroblast growth factor 19 (FGF19); they also influence cholesterol metabolism and systemic energy expenditure [40].

Bile acids function as substrates for biotransformation by the gut microbiota, thereby influencing the composition and behavior of the microbial community within the gastrointestinal lumen. For example, the microbiota promotes biosynthesis of secondary bile acids [41,42], including deoxycholic acid and lithocholic acid. Importantly, the consumption of a high-fat diet substantially increases the levels of secondary bile acids within both fecal matter and systemic circulation [43,44,45].

The metabolism of the gut microbiota affects both the bioavailability and bioactivity of bile acids; these properties influence the metabolic processes in which bile acids participate, as well as the development of obesity [46]. Studies in animal models have demonstrated that gut microbiota-mediated regulation of bile acid synthesis and metabolism can influence body weight and fat distribution; the inhibition of bile acid synthesis leads to improved glucose tolerance [47] and weight loss [48]. Nierop et al. revealed that in obese and insulin-resistant individuals, the induction of weight loss by a very low-calorie diet led to increased postprandial deoxycholic acid levels and reduced resting energy expenditure [49]. Those findings highlighted the important roles of secondary bile acids in regulating energy metabolism and body weight via receptor activation, which decreases glucose and lipid synthesis, improves insulin sensitivity, and reduces energy storage [50,51,52,53].

### 3.2. SCFAs

SCFAs are produced in the gastrointestinal tract via microbial fermentation of carbohydrates; acetate, propionate, and butyrate are the primary SCFAs produced by this method [54]. The production of SCFAs is influenced by changes in gut microbiota structure and function; a decrease in the number of butyrate-producing bacteria is closely associated with increased energy storage and weight gain in both animal models and humans [55]. Butyrate regulates energy metabolism by interacting with various metabolic pathways; it can stimulate energy expenditure while mitigating adipocyte size via lipogenesis inhibition and the promotion of lipolysis-related genes [56]. Moreover, butyrate can enhance insulin sensitivity and glucose homeostasis by activating adenosine monophosphate-activated protein kinase (AMPK) and repressing inflammation [57].

SCFAs provide energy for the epithelial cells and influence the immune function of the mucosa, by regulating the pH and the production of mucus in the intestinal lumen. Increased mucus production is usually inferred from increased expression of the MUC2 gene encoding mucin 2, and SCFAs, particularly butyrate, stimulate MUC2 gene expression through selective acetylation/methylation of the MUC2 histone, thereby promoting mucus production [58]. Furthermore, SCFAs can enter the systemic circulation, then directly affect metabolism and function in peripheral tissues [58,59,60]. Although SCFAs serve as an immediate source of additional energy, increased levels of SCFAs are presumed to support whole-body energy regulation via reduced production of hepatic glucose and lipids [55,61].

SCFAs regulate appetite and food intake by influencing communication between the gastrointestinal tract and the central nervous system [58,59,60,62]. One of these mechanisms involves the stimulation of colonic cells that express free fatty acid receptor 2 (FFAR2) and free fatty acid receptor 3 (FFAR3) [63], which are receptors for SCFAs, especially acetate and propionate. Upon activation, these cells secrete hormones that promote satiety, such as PYY and GLP-1.

In rodents, acute or chronic administration of SCFAs can mitigate weight gain and induce weight loss [58]. For example, Hattori et al. [64] found that oral administration of acetate stimulated energy expenditure in mice compared to a water control group, and Kimura et al. [65]. reported that intraperitoneal administration of propionate increased oxygen consumption in mice compared to the phosphate group. These studies show that SCFA supplementation dramatically increases energy expenditure in rodents. Regarding the chronic perspective, Den Besten et al. [66] found that the addition of sodium acetate, sodium propionate, or sodium butyrate to a high-fat diet increased energy expenditure in mice after 10 weeks during both the 12 h day and night periods. Sahuri-Arisoylu et al. [67] also found an increase in energy expenditure on a high fat diet after 6 weeks of intraperitoneal administration of nanoparticle-derived sodium acetate during the 12 h day and night periods. Some researchers have studied the metabolism of butyrate in both acute and chronic ingestions. Interestingly, Li et al. [28] reported a reduction in energy intake following both the acute and chronic intragastric administration of butyrate, but this reduction disappeared when the mode of ingestion was changed to intravenous administration. The likely reason for this is that intragastric administration allows butyrate to reach its site of action and to interact with intestinal receptors and/or metabolism, which is not achieved with the periphery. Despite the importance of SCFAs in regulating energy metabolism and body weight, the current literature regarding SCFAs in human health is predominantly focused on exogenous fiber supplementation and endogenous SCFA production. Thus far, the findings are inconclusive because of difficulties in measuring SCFA production [58].

### 3.3. Amino Acids

The gut microbiota is a major source of various metabolites, including amino acids. The diverse effects of these compounds on energy balance and metabolic homeostasis are mediated by multiple mechanisms. For example, gut microbiota-derived amino acids have been consistently implicated in alterations of energy metabolism, regulation of body weight, and accumulation of adipose tissue. Notably, phenylalanine is converted to tyrosine, which serves as a precursor to catecholamines such as epinephrine and norepinephrine. By enhancing energy expenditure, promoting fat oxidation, and activating brown adipose tissue, these catecholaminergic molecules may contribute to obesity prevention [68]. 

Tryptophan, an essential amino acid, is utilized in protein synthesis; it also plays a key role in the gut microbiota-mediated regulation of body weight and metabolism [69]. Tryptophan and its metabolites can transmit signals locally, especially in the intestinal mucosa; they can also transmit signals to other organs, such as the brain [70]. Some tryptophan metabolites, which are derived from microbial degradation, have demonstrated robust effects on appetite regulation in various experimental models [71,72,73]. These metabolites include tryptamine, indole-3-acetic acid, and 3-indole-propionic acid, all of which serve as ligands for the aryl hydrocarbon receptor (AhR), a naturally occurring receptor for aryl hydrocarbons [74]. AhR, a sensor for environmental and physiological signals, has strong effects on intestinal barrier function, immune response, and metabolism [75,76]. Altered levels of tryptophan-derived metabolites have been detected in stool samples from individuals with metabolic syndrome; these changes were associated with reduced AhR activity [74]. Impaired microbial synthesis of AhR ligands leads to the deterioration of mucosal barrier integrity and diminished secretion of GLP-1, with subsequent contributions to the onset of metabolic syndrome, type 2 diabetes mellitus (T2DM), high body mass index, and hypertension [74].

The intestinal microbiota can synthesize branched-chain amino acids, namely leucine, isoleucine, and valine. These amino acids have been associated with the onset of insulin resistance, obesity, and subsequent metabolic disorders [7]. Some studies have shown that fasting serum BCAA levels are higher in people with type 2 diabetes than in healthy individuals [77], and that BCAA levels decrease after weight loss surgery or dietary supplementation with BCAA in obese individuals or rats [78]. These findings suggest that BCAAs or their breakdown products may play a causal role in metabolic disorders [79,80], although the exact mechanisms are not fully understood. The gut microbiota may also affect the blood levels of BCAAs by altering the expression or activity of genes involved in BCAA biosynthesis or transport. Some bacteria, such as Prevotella copri and Bacteroides vulgatus, have been found to have a high potential for BCAA biosynthesis and inward transport, and to be more abundant in people with insulin resistance than in healthy individuals [7]. These bacteria may directly affect host metabolism by increasing the production or uptake of BCAAs in the gut. This hypothesis has been tested in mice fed a high-fat diet and infected with P. copri, which resulted in increased serum BCAA levels, insulin resistance, and glucose intolerance [7].

### 3.4. Other Gut Microbiota-Derived Metabolites

Extensive research concerning the roles of gut microbiota-derived metabolites in obesity has been performed [81,82,83]. Some of these metabolites include phenolic acids, lipopolysaccharides, ethanol, and microbial-derived protein fermentation products. These metabolites contribute to obesity by affecting insulin sensitivity, altering energy balance, and causing low-grade inflammation. For example, phenolic acids can modulate adipocyte differentiation and lipolysis [82], lipopolysaccharides can induce inflammation and impair glucose uptake in adipose tissue, ethanol can increase hepatic lipid accumulation and insulin resistance [82], and protein fermentation products can activate pro-inflammatory pathways and reduce energy expenditure [82]. These findings highlight the need for continued research into the complex mechanisms by which gut microbiota-derived metabolites contribute to the development of obesity.

## 4. Microbiota–Target Tissue Interactions in Obesity

Multiple studies have demonstrated correlations between disruptions in the taxonomic and functional properties of the gut microbiota and various histopathological phenotypes. The human gut microbial gene pool has provided insights concerning the wide range of functions exhibited by the gut microbiota, which functions as an extensive chemical factory with the ability to synthesize a diverse range of compounds that promote microbial and host survival [9,84,85,86]. Gut microbiota-derived metabolites exert extensive effects, both locally and in distant tissues; they may also facilitate microbial translocation into various tissues and organs, including human adipose tissue, by increasing intestinal permeability. Nevertheless, the host metabolism-related effects of microbes and microbial DNA in peripheral tissues remain unclear. Some members of the gut microbiota and their metabolites that are associated with host tissues (e.g., adipose tissue, intestinal epithelial cells, and muscle) are discussed below.

### 4.1. Adipose Tissue

Obesity is primarily characterized by irregularities in adipose tissue, which mainly comprise atypical fat distribution and adipose tissue dysfunction. Abnormal fat distribution is defined as excessive accumulation of visceral adipose tissue in the intraperitoneal and retroperitoneal regions, as well as ectopic fat deposition in non-physiological sites (e.g., liver, pancreas, heart, and skeletal muscle). Adipose tissue dysfunction involves impaired adipogenesis, adipocyte hypertrophy, and anomalous lipid metabolism [87]. The pathogenesis underlying adipose tissue dysfunction is related to the perpetuation of a vicious cycle [88] characterized by macrophage infiltration and proinflammatory polarization (M1 polarization), which triggers a cascade of inflammatory pathways that have detrimental effects on insulin signaling [89]. Moreover, the combination of aberrant adipokine production and regulation increases the likelihood of adipose tissue dysfunction.

The interaction of specific dietary factors, such as a high-fat diet and certain gut microbiota, can interfere with adipose tissue function and lead to severe adipose tissue dysfunction [90]. Tran et al. showed that Western diet-induced dysbiosis can cause adipose tissue inflammation in mice, as demonstrated by an increase in typical proinflammatory M1 macrophages and a decrease in anti-inflammatory M2 macrophages within adipose tissue [91]. They also showed that the ablation of gut microbiota could reduce this inflammatory response within adipose tissue. Additionally, experimental knockdown of the Toll-like receptor (TLR) signaling protein myeloid differentiation primary response protein 88 (MyD88) yielded a phenotype similar to the phenotype caused by microbiota ablation, supporting the notion that Western diet-induced adipose tissue inflammation does not result from lipid accumulation; instead, the microbiota and/or its metabolites activate innate immune signaling pathways, which lead to inflammation. Although gut microbiota-derived metabolites were not examined in the study by Tran et al., the results indicated that gut microbiota and their metabolites induce adipose tissue inflammation through a TLR-mediated inflammatory response. Previous research concerning LPS-mediated activation of TLRs revealed associations with the development of obesity [92], suggesting a robust relationship between gut microbiota-derived LPS and adipose tissue inflammation [93].

**Figure 1:**
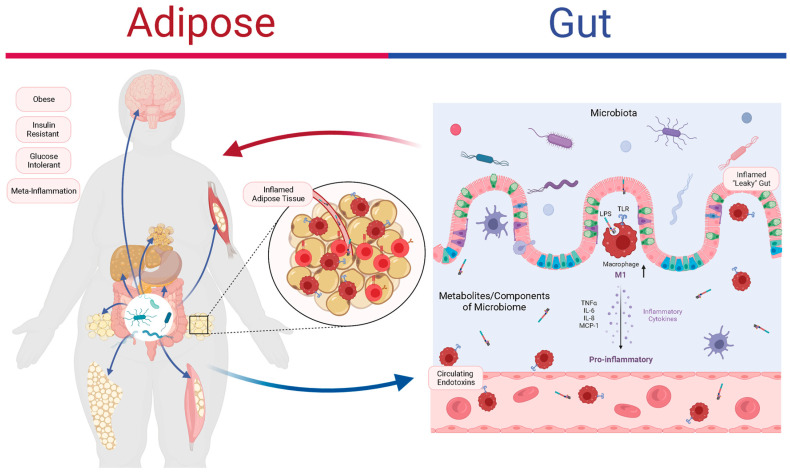
The role of the gut microbiota and inflammation in adipose tissue dysfunction and obesity. Obesity is associated with abnormal fat distribution and adipocyte dysfunction, which result in chronic inflammation and insulin resistance. The gut microbiota can affect the intestinal barrier function, the immune system, and the adipose tissue metabolism. The gut microbiota produces various metabolites, such as lipopolysaccharides (LPS), that can activate Toll-like receptors (TLRs) on macrophages and intestinal epithelial cells (IECs), triggering inflammatory pathways that impair insulin signaling and promote adipose tissue inflammation. The adipose tissue secretes hormones and cytokines, such as tumor necrosis factor-alpha (TNF-α), and interleukin-6 (IL-6), that can modulate the gut microbiota’s composition and function. The interactions between the gut microbiota and the adipose tissue are complex and bidirectional, forming a vicious cycle that leads to adipose tissue dysfunction and obesity. Figure created using BioRender.com.

Additionally, the results of recent studies are consistent with the notion that gut microbiota dysbiosis represents an early indication of inflammation and obesity [94]. The gastrointestinal system interacts with the dietary components involved, which disrupts the gut microbiota’s balance and alters the secretion profiles of gut peptides [16]. These disruptions trigger an inflammatory response in the intestinal mucosa, causing damage to the epithelial barrier and enhancing LPS entry into the systemic circulation. LPS and saturated fatty acids can activate TLRs, which are receptors that recognize microbial molecules, on macrophages or intestinal epithelial cells [95]. This leads to low-grade systemic inflammation. TLRs are also expressed in adipose tissue, where they can be activated by LPS and induce the secretion of inflammatory cytokines, such as TNF-α, IL-6, IL-8, and MCP-1, by macrophages and adipocytes [96,97,98,99]. These cytokines can attract more inflammatory cells to adipose tissue and worsen the inflammation. Chronic inflammation can also affect the gut microbiota and cause dysbiosis [100]. For example, obesity decreases the abundance of *Akkermansia muciniphila* in mice, with a concomitant increase in circulating C-reactive protein [101]. IL-36, which stimulates *Akkermansia* proliferation, plays a key role in protecting against obesity and metabolic disorders [102]. Overall, the interactions between the gut microbiota and inflammation are highly complex; they often involve gut bacteria-driven immune activation, systemic low-grade inflammation leading to adipose inflammation, and the resultant adipose tissue dysfunction that perpetuates further systemic inflammation, insulin resistance, and (ultimately) obesity (Figure 1).

### 4.2. Intestinal Epithelium

Gut barrier dysfunction and gut microbiota dysbiosis are linked to a diverse array of pathological conditions, including obesity, T2DM, and inflammatory bowel disease. Intestinal epithelial cells play a key role in maintaining the health of the gut microbiota. These cells form a physical barrier between the gut microbiota and the underlying tissues, preventing the migration of harmful bacteria and small molecules into the systemic circulation. Tight junctions between the epithelial cells function as gatekeepers that regulate the entry of nutrients and other substances into the intestinal epithelium. Dysregulation involving these junctions can lead to gut dysbiosis and intestinal permeability, triggering various pathological conditions. Thus, the preservation of epithelial cell integrity is essential to maintain gastrointestinal health and prevent gut microbiota-related diseases.

The Firmicutes phylum, including the *Lactobacillus* genus, has been linked to obesity and T2DM [103]. However, some genera in this phylum (e.g., *Lactobacillus paracasei* and *Lactobacillus plantarum*) can help to prevent weight gain [104,105]. Decreased relative abundances of *Bacteroides* and *Bifidobacterium*, as well as the butyrate-producing *Faecalibacterium prausnitzii* and *Roseburia intestinalis*, have been associated with obesity [106,107,108]. In T2DM patients, bariatric surgery increases the relative abundance of *F. prausnitzii* while improving glucose homeostasis, low-grade inflammation, and intestinal epithelial permeability [14,109]. *F. prausnitzii* is a key producer of butyrate, which serves as an important energy source for intestinal epithelial cells [110]. Multiple butyrate-mediated mechanisms (e.g., mucin synthesis [111], reorganization of tight junctions, and upregulation of occludin and Zonula occludens protein 1 [112,113]) can reduce local inflammation and improve intestinal barrier permeability. The species *Bacteroides vulgatus* and *Bacteroides dorei* may provide benefits for T2DM patients by increasing Zonula occludens protein 1 (ZO-1) expression and enhancing epithelial barrier function [114]. These bacteria produce bacteriocins, a class of proteins that inhibit the growth of specific microbes and could reduce the abundance of harmful species [115]. Additionally, a mucin-degrading species in the Verrucomicrobia phylum, *Akkermansia muciniphila*, colonizes the intestinal mucus layer and enhances intestinal barrier integrity; these effects are mediated by promotion of mucin production (direct mechanism) [116] and by interactions with other bacteria (indirect mechanism) [117,118]. *A. muciniphila* has been linked to reductions in wasting, insulin sensitivity, and low-grade inflammation [117,119]. Furthermore, the relative abundances of Gram-negative bacteria (primary producers of LPS) are altered in obesity; the dysregulated gut microbiota tends to shift towards the more proinflammatory LPS from *Proteus* spp., rather than the less potent LPS from *Bacteroides* spp. [120,121]. Gut microbiota dysregulation can lead to increased levels of proinflammatory endotoxins, thereby exacerbating low-grade inflammation by enhancing transepithelial permeability; these changes promote the circulation of proinflammatory LPS [118,122].

The induction of low-grade inflammation in the presence of a damaged intestinal barrier has been attributed to the proinflammatory LPS produced by Gram-negative bacteria [93]. Under normal circumstances, the intestinal epithelium provides protection against LPS translocation; however, models of diet-induced obesity and T2DM (e.g., *db*/*db* mice) display increased levels of circulating LPS and subsequent inflammation [93]. Moreover, mice with diet-induced obesity and *db*/*db* mice exhibit increased transepithelial permeability and disrupted ZO-1 distribution in the intestinal mucosa, compared with lean control mice [123]. This significant alteration of intestinal barrier permeability has been linked to decreased levels of the porosity-regulating tight junction proteins ZO-1 and occludin [90,124]. Furthermore, studies in humans have revealed higher gastrointestinal epithelial permeability among individuals with obesity [125]. However, despite the potential role of obesity in intestinal barrier dysfunction, hyperglycemia is considered the predominant factor that promotes intestinal barrier disruption in obese and T2DM mice [126]. This disruption has been directly linked to hyperglycemia and can be remedied by glycemic control [126]. Similarly, poor glycemic control in humans has been linked to increased entry of microbial products into the bloodstream [126]. The severity of hepatic steatosis in obese individuals has also been linked to an increase in intestinal permeability [123,127]. These observations suggest a strong linkage between changes in intestinal barrier function and the onset of obesity-related complications.

### 4.3. Muscle

Skeletal muscle, which comprises approximately 40% of the human body mass, is responsible for numerous critical functions including thermoregulation and the modulation of glucose/amino acid metabolism. Although it is physically distinct from the gut, skeletal muscle is influenced by gut-derived signals that arise from interactions between gut microbiota and host tissue; these interactions involve microbes, metabolites, gut peptides, LPS, and ILs. These signals form a link between gut microbiota activity and skeletal muscle function; modulation of these signals influences systemic or tissue inflammation and insulin sensitivity, helping to regulate muscle function. Disruptions in gut microbiota composition can lead to muscle atrophy, weakness, and poor exercise performance. Additionally, some microbial metabolites, such as SCFAs, have direct effects on muscle health and function, highlighting the complex interactions between gut microbiota and muscle physiology. Thus, the gut microbiota represents a promising new target for the prevention and treatment of muscle-related diseases.

Considering the diverse array of gut microbiota-derived metabolites, research has focused on the potential for SCFAs to mediate interactions among the gut microbiota, gastrointestinal physiology, and muscle insulin sensitivity. Comparative analyses have revealed that exercise interventions are associated with greater abundances of SCFA-producing microbial taxa, compared with the abundances in individuals with sarcopenia. SCFAs are primarily generated by microbial anaerobic fermentation of nondigestible dietary fibers, mainly within the distal ileum and colon. Acetate, propionate, and butyrate comprise the predominant SCFA profile within the colon, totaling more than 95% of the total SCFA content. Upon entry into enterocytes, butyrate drives the citric acid cycle through acetyl-CoA, satisfying up to 60–70% of colonocyte metabolic needs [55]. The remaining SCFAs are transported through the portal vein to the liver, which absorbs up to 80% of the available propionate and 40% of the available acetate for subsequent utilization in gluconeogenesis [55,128]. Finally, a small subset of SCFAs, predominantly acetate, is transported to skeletal muscle.

SCFAs are important for maintaining glucose and lipid homeostasis, regulating inflammation, and establishing connections between the gut and distant tissues [55,129]. There is empirical evidence that SCFA supplementation can enhance muscle mass and strength, particularly in germ-free and antibiotic-treated rodents [130,131,132,133]. Notably, acetate supplementation (via dietary intake or subcutaneous injection) enhances glucose uptake and glycogen content while reducing lipid accumulation in rat skeletal muscles [134]. In mice, oral supplementation of butyrate protects against oxidative stress and loss of muscle mass; it also increases mitochondrial function and increases the number of type I fibers in skeletal muscles [133,135]. Although SCFAs may provide metabolic benefits for skeletal muscles (e.g., acetate is present in peripheral blood), butyrate and propionate may only be present in small amounts in peripheral blood. However, the effects of SCFAs may be indirectly achieved via the secretion of GLP-1, a gut hormone that stimulates insulin secretion, glucose storage in the liver, and glucose uptake in skeletal muscles [135]. Moreover, SCFAs can increase blood flow to muscles and exert anti-inflammatory effects through epigenetic mechanisms [136]. Similar to propionate, succinate serves as a substrate for gluconeogenesis. The detection of enterocyte-derived glucose—produced from succinate—in the portal vein can increase satiety, energy expenditure, glucose tolerance, and insulin sensitivity [137,138].

### 4.4. Other Target Organs or Tissues

In addition to its effects on adipose tissue, intestinal epithelium, and muscle, the gut microbiota interacts with other physiological targets and organs during the development of obesity. The gut microbiota is reportedly involved in the regulation of the hypothalamic–pituitary–adrenal axis, a key factor in energy balance and metabolism [139]. This axis is regulated by a complex network of signaling pathways involving the central nervous system, gut microbiota, and other peripheral tissues, including the liver and adipose tissue.

Recent evidence suggests that the gut microbiota helps to modulate the gut–liver axis, a key regulatory component in metabolic homeostasis. Specifically, the liver can be influenced by gut microbiota-derived metabolites, which exert downstream effects on hepatic metabolism, gene expression, and insulin sensitivity [140,141].

Moreover, the gut microbiota affects the cardiovascular system by influencing blood pressure, lipoprotein metabolism, and systemic inflammation [142]. Additionally, the gut microbiota has regulatory effects on the immune system; gut microbiota dysregulation has been associated with the onset of obesity-related inflammatory and metabolic disorders [143].

## 5. Study Strengths and Limitations

This review provides an updated overview of the current literature on the gut microbiota and obesity, covering various aspects of the gut microbiota–host tissue interactions and the gut microbiota-derived metabolites, and emphasizing the roles of adipose tissue microenvironment interactions in the onset of obesity. However, this review has some limitations that need to be acknowledged. One limitation is that most of the studies included are based on rodent models, which may not fully reflect the human situation. Another limitation is that it does not adequately address the effects of various dietary factors on the gut microbiota and obesity. Future research should investigate how different components of the Western diet, such as trans-fatty acids, easily digestible carbohydrates, antibiotic residues, hormones, preservatives, or pesticides, influence the gut microbiota and obesity. 

## 6. Conclusions

The cumulative findings of numerous studies highlight the complex and multifaceted link between intestinal microflora and obesity. These studies have demonstrated that gut microbiota dysregulation influences energy equilibrium and can contribute to the onset of obesity. Furthermore, these studies have identified potential mechanisms by which the gut microbiota and its metabolites affect the onset of obesity, including the production of endogenous metabolites, regulation of systemic inflammation, and modulation of the adipose tissue microenvironment.

Future research in this area is needed to elucidate the mechanisms that underlie the relationships of the gut microbiota and its metabolites with obesity. Such studies will contribute key insights concerning the viability of microbiota-centered interventions for human health. Accordingly, continued investigation of the interactions between the gut microbiota and its metabolites in obesity has important implications for efforts to manage the growing obesity pandemic.

## Figures and Tables

**Table 1:** Obesity-associated changes in the gut microbiota.

Upregulated	Downregulated	Reference
Firmicutes; Firmicutes to Bacteroidetes ratio	Bacteroidetes;	Turnbaugh PJ et al., Nature. (2006) [2]; Hildebrandt MA et al., Gastroenterology. (2009) [15]
*Clostridium cluster XIVa*	*Lactobacillus*; *Clostridium cluster IV*; *Faecalibacterium prausnitzii*; *Archaea*	Remely M et al., Benef Microbes. (2015) [13]
Mollicutes	*Akkermansia*; *Faecalibacterium*; *Oscillibacter*; *Alistipes*	Thingholm LB et al., Cell Host Microbe. (2019) [10]
*Escherichia-Shigella*	*Faecalibacterium*	Anhê FF et al., Nat Metab. (2020) [9]
	*Bacteroides*; *Prevotella*	Furet JP et al., Diabetes. (2010) [14]
	*Oscillospira*	Gophna U et al., Environ Microbiol. (2017) [12]
	*Bacteroides thetaiotaomicron*	Liu R et al., Nat Med. (2017) [8]
*Bacteroidales*; *Clostridiales*		de La Serre CB et al., Am J Physiol Gastrointest Liver Physiol. (2010) [16]
*Desulfovibrionaceae*		Zhang C et al., ISME J. (2010) [17]
Fibrobacteres		Geurts L et al., Front Microbiol. (2011) [11]

**Table 2:** Functions of microbial metabolites in obesity.

	Metabolites	Function	Reference
Amino acid	Leucine, Isoleucine and Valine (BCAAs)	Mitigate insulin resistance	Mollard RC et al., Am J Clin Nutr. (2014) [22]
	Glutamine	Reduce inflammation in adipocytes	Petrus P et al., Cell Metab. (2020) [23]
	L-Serine	Increase brown adipose tissue activity	López-Gonzales E et al., Nutrients. (2022) [24]
Bile acids	Deoxycholic acid (DCA);	Inhibit adipose thermogenesis	Schroeder BO et al., Nat Med. (2016) [25]
	Taurochenodeoxycholic acid (TCDCA); Lithocholic acid (LCA)	Induces browning of white adipose, enhances insulin sensitivity, and improves liver metabolism	Pathak P et al., Hepatology. (2018) [26]
	Glycoursodeoxycholic acid (GUDCA)	Hyperglycemia	Sun L et al., Nat Med. (2018) [27]
Short-chain fatty acids	Butyrate	Promote fat oxidation, activate brown adipose tissue	Li Z et al., Gut. (2018) [28]; van Deuren T et al., Obes Rev. (2022) [29]
	Acetate	decrease lipolysis, increase energy expenditure	Hernández MAG et al., Nutrients. (2019) [30]
	Propionate	Improve insulin resistance	Chambers ES et al., Gut. (2019) [31]
Other metabolites	Indole; indoxyl sulfate; indole-3-carboxylic acid (I3CA)	Regulate energy expenditure, increase insulin sensitivity	Virtue AT et al., Sci Transl Med. (2019) [32]
	10-hydroxy-cis-12-octadecenoic acid (HYA)	Induces browning of white adipose	Kim M et al., FASEB J. (2017) [33]
	Succinic acid	Induce brown adipocyte thermogenesis	Mills EL et al., Nature. (2018) [34]
